# Candidate Genes for Aggressiveness in a Natural *Fusarium culmorum* Population Greatly Differ between Wheat and Rye Head Blight

**DOI:** 10.3390/jof4010014

**Published:** 2018-01-16

**Authors:** Valheria Castiblanco, Hilda Elena Castillo, Thomas Miedaner

**Affiliations:** State Plant Breeding Institute, University of Hohenheim, 70599 Stuttgart, Germany; vcastiblanco@gmail.com (V.C.); hildaelenac@gmail.com (H.E.C.)

**Keywords:** association mapping, aggressiveness, candidate gene, cutinase, Fusarium head blight (FHB), quantitative trait loci (QTL), single-nucleotide polymorphism (SNP)

## Abstract

*Fusarium culmorum* is one of the species causing Fusarium head blight (FHB) in cereals in Europe. We aimed to investigate the association between the nucleotide diversity of ten *F. culmorum* candidate genes and field ratings of aggressiveness in winter rye. A total of 100 *F. culmorum* isolates collected from natural infections were phenotyped for FHB at two locations and two years. Variance components for aggressiveness showed significant isolate and isolate-by-environment variance, as expected for quantitative host-pathogen interactions. Further analysis of the isolate-by-environment interaction revealed the dominant role of the isolate-by-year over isolate-by-location interaction. One single-nucleotide polymorphism (SNP) in the cutinase (*CUT*) gene was found to be significantly (*p* < 0.001) associated with aggressiveness and explained 16.05% of the genotypic variance of this trait in rye. The SNP was located 60 base pairs before the start codon, which suggests a role in transcriptional regulation. Compared to a previous study in winter wheat with the same nucleotide sequences, a larger variation of pathogen aggressiveness on rye was found and a different candidate gene was associated with pathogen aggressiveness. This is the first report on the association of field aggressiveness and a host-specific candidate gene codifying for a protein that belongs to the secretome in *F. culmorum*.

## 1. Introduction

Fungi are the most important pathogens that attack cereal crops in Central Europe. Among them, the genus *Fusarium* is a worldwide threat to many agricultural crops and commodities reducing not only the yield, but contaminating the grain with mycotoxins [1]. They induce seedling blight, foot and root rot, and head blight in the field. Fusarium head blight (FHB) is one of the most common and harmful diseases that affect all small-grain cereals and some forage grasses worldwide [1]. From infected ears, about 13 different species can be isolated, among them *Fusarium graminearum*, *F. culmorum*, and *F. avenaceum* are the most common in Europe [1]. Outbreaks of FHB result in yield losses and quality reduction, while mycotoxins produced by the pathogen lead to contamination of grain. There is substantial evidence of risks to human and animal health posed by FHB mycotoxins [2]. An estimated $7.67 billion loss was caused by FHB in wheat and barley production alone in the period between 1993 and 2001 in the USA [3].

*F. culmorum* (W.G. Smith) Sacc., firstly described in 1892, is a soil-borne pathogen and the principal origins of inoculum are crop residues containing fungal mycelium and long-living chlamydospores in the soil [4]. Main risk factors for FHB infection are maize as previous crop, reduced soil management, especially no tillage, a susceptible wheat cultivar, and favorable weather conditions. Cereal plants are most vulnerable to FHB infection during flowering till the soft dough stage. Wet and warm weather in the periods of crop anthesis and maturation can increase the risk of development of FHB [5]. When the macroconidia reach the ear, they germinate and the fungus can grow into cereal florets either passively by natural openings, for example, the stomata [6], or actively by direct penetration of the cuticle and cell walls. This is facilitated by a great range of hydrolyzing enzymes such as cutinases, cellulases, pectin lyases and xylanases, which are released by the fungus during the penetration process [7].

*F. culmorum* and *F. graminearum* belong to the category of hemibiotrophic pathogens. Hemibiotrophs present a short biotrophic phase throughout the primary phase of infection and then switch to necrotrophy with secretion of mycotoxins and enzymes for degradation of host cell walls [8,9]. Trichothecenes are mostly produced by proteins and regulators encoded by the *TRI* genes located at the trichothecene gene cluster [2,10]. Among the trichothecenes, deoxynivalenol (DON) is the most common mycotoxin, but also nivalenol is produced by some isolates of both species. Additionally, all isolates produce zearalenone, a compound exhibiting oestrogenic properties in mammals.

From the host perspective, the genetic basis of FHB resistance in cereals has been explored in a large number of studies that observed a quantitative inheritance [5,7,8,9]. This type of resistance is controlled by many genes, each with a small phenotypic effect and affected by the environment (locations, years). Quantitative resistance is not race specific, i.e., the same plant genotypes display an equivalent ranking against all pathogen isolates [11] and the resistance should be less prone to pathogen adaptation and, hence, more durable.

A key factor that determines parasitic fitness of an isolate is aggressiveness that describes the quantitative pathogenicity and should, hence, be quantitatively measured [11]. Aggressiveness is frequently evaluated by directly assessing epidemic rates [12], and reflects several basic quantitative traits of the fungal life cycle, such as infection efficiency, sporulation, sizes of the lesion, and toxin production [13]. Mycotoxin production and their effects in aggressiveness have been studied in detail in Fusarium species. Cumagun & Miedaner [12] reported a positive correlation (*r* = 0.7, *p* < 0.01) between aggressiveness and DON production using 50 isolates of *F. graminearum*. A similar outcome was reported for 100 *F. culmorum* isolates in wheat (*r* = 0.67, *p* < 0.001 [14]).

In contrast to a large number of studies on host resistance, studies on the genetic basis of fungal aggressiveness are very limited. Therefore, it is necessary to close this knowledge gap about genetic and environmental determinants of aggressiveness to make assumptions on the possible adaptation of the pathogens to host resistance. In the case of *F. culmorum* and *F. graminearum*, there is a high probability that many genes are associated with aggressiveness but the precise number and interaction between them are still to be established.

In the related species *F. graminearum* with frequent sexual recombination the development of mapping populations is possible. In a study [15] using this approach, two quantitative trait loci (QTL) for aggressiveness linked to the *TRI5* locus were identified. Unfortunately, this approach is not an option for *F. culmorum* because no teleomorph has been identified yet [16].

With the advantage of having the complete genome sequence of *F. graminearum* with four chromosomes comprising 36.6 Mbp [17,18], it is now possible to use other approaches such as candidate gene association mapping, a powerful tool to identify functional polymorphisms related with aggressiveness [19]. This requires the use of a panel of unrelated isolates that show a wide range of variation. Candidate genes are one option for association mapping. This approach is relatively economical and quick to perform when the full genomic sequence of the pathogen is available. It begins with the selection of a putative candidate gene according to its importance in the mechanisms of the trait being examined. Hence, previous knowledge about gene function is required [19]. The second step is to detect polymorphisms within the gene, which can affect the gene regulation or its product [20]. Finally, the polymorphisms in nucleotide diversity are verified for their association with phenotypic changes. With candidate gene association mapping, SNPs in three genes (TRI1, *MetAP1*, *Erf2*) were significantly associated with aggressiveness in *F. graminearum* in wheat [21]. An alternative is the classical association mapping where the whole genome is saturated by molecular markers and distinct peaks show associations to phenotypic values. This has also been adopted in *F. graminearum* [22] and resulted in the identification of seven and five genes for aggressiveness and DON production, respectively. However, the function of the associated genes in relation to pathogenicity is not known.

*F. culmorum* has a broad host spectrum including all small-grain cereals [23]. In Europe, wheat and rye are the most widely distributed bread-making cereals. Bread wheat (*Triticum aestivum* L.) was grown on about 62.5 million hectares in 2016, rye (*Secale cereale* L.) across 3.6 million hectares [24]. Both cereals are mainly used as winter crops and have a very similar growth pattern, although winter rye is flowering about three weeks earlier than bread wheat. While bread wheat is a self-pollinating crop with homozygous line cultivars, rye is an outcrossing crop with a heterogeneous type of cultivars.

The goals of this research were to (i) untangle the relative importance of the components explaining the variance of aggressiveness measured in field experiments across two replications, two locations and two years, with an experimental, genetically homogeneous winter rye genotype as a host; (ii) compare the phenotypic information from rye and wheat; (iii) evaluate the association of SNPs in the candidate genes with *F. culmorum* aggressiveness quantified with two different Data sets (Table S1) using (a) only rye as host across two locations and two years (2015, 2016, Data set 1) and (b) the phenotypic information from rye and wheat across two locations in 2015 (Data set 2).

## 2. Materials and Methods

One hundred isolates of *F. culmorum* from a collection described in a prior study were used [25] (Table 1). They belong to four different field populations, one from Russia and three from Germany, one Syrian transect population and an international collection of the State Plant Breeding Institute, University of Hohenheim. Isolates were acquired from ears displaying observable FHB symptoms in the field.

Mycelial disks of Fusarium isolates were grown on synthetic nutrient-poor agar (SNA) medium and transferred in 2.5 mL Eppenmeyer tubes in distilled water at 6 °C for storage. One agar plug out of the stored isolates was placed in Erlenmeyer flasks with 400 mL of the SNA medium and incubated under constant shaking at 110 rpm and UV light for stimulation of sporulation during 1 week at 22–25 °C [26]. With a hemacytometer, the spores were counted for each isolate, from which the concentration of spores was calculated and aliquots frozen at −80 °C were prepared. Before application, the samples were thawed in water at 20 or 40 °C [27], and brought to a final concentration of 2 × 10 ^5^ spores.

The spores were inoculated on the rye heads at full flowering with a manual atomizer and 100 mL suspension per square meter. A tractor was used to generate a stable air pressure of 3 bars to guarantee the even application of the spores on rye heads across the plot.

A susceptible, cytoplasmic-male sterile single cross of winter rye was used as host across the whole experiment (*Secale cereale* L., “L2177-P×L2184-N”, HYBRO Saatzucht GMBH & Co., KG, Schenkenberg, Germany). The trial was made in two locations: Oberer Lindenhof (OLI, altitude 700 m, longitude 9°18′12′′ E, latitude 48°28′26′′ N) and Hohenheim (HOH, altitude 400 m, longitude 9°12′58′′ E, latitude 48°42′50′′ N) in two years (2015 and 2016). For comparison, previously reported phenotypic data from wheat were used [14], corresponding to the measurements of aggressiveness of the same 100 *F. culmorum* isolates tested on a moderately susceptible winter wheat cultivar (“Inspiration”, KWS LOCHOW GMBH, Bergen, Germany) with the same experimental conditions at the same locations and experimental design in 2014 and 2015. Comparison between crops was restricted to 2015, because only in this year the experiments were placed on the same field as split-plot design with crops as main plots and isolates as subplots. Means of annual temperature at OLI and HOH in 2015 were 8.88 °C and 10.86 °C and in 2016 were 8.5 °C and 10.12 °C, respectively. The mean precipitation at OLI and HOH were 709.8 mm and 492.1 mm in 2015 and 779.3 mm and 595.4 mm in 2016.

Seeds were grown in two-row plots with 1 m length and 0.42 m width. To decrease the drifting or secondary spore dispersal and avoid possible interference among plots, a chessboard-like design was used to arrange the plots that were bordered by long-strawed rye. The latter was a mix of two population cultivars: “Dukato” (Hybro Saatzucht GmbH & Co., KG) and “Conduct” (KWS LOCHOW GMBH) to secure pollination. Plots were sown with 220 kernels m^−2^.

The experiment was arranged according to an alpha-lattice design with two replications per environment and an incomplete block size of ten plots. The randomization of genotypes was done by PLABPLAN (Version 1E, University of Hohenheim (350a), 70599 Stuttgart, Germany) within the program package PLABSTAT [28].

The ratings started with the initiation of symptoms about two weeks after inoculation and continued in 2 to 5 days intervals until the start of yellow ripening. Typical symptoms are the prematurely bleaching of infected cereal spikelets while the non-infected part of the head is still green [1,16]. In inoculation experiments, several to many adjoining spikelets are often affected by aggressive isolates under favorable weather conditions. In extreme, the whole head could turn white. FHB aggressiveness was evaluated visually three to five times as the percentage of infected spikelets per plot. This result sums up the percentage of infected spikes per plot and the percentage of infected spikelets per spike in one rating. For further calculations, the arithmetic mean of the ratings (=mean FHB ratings) was used.

The phenotypic data from each environment were separately screened for outlier detection with the Bonferroni-Holm method with re-scaled MAD standardized residuals as suggested by Bernal-Vasquez [29]. Additionally, the results from the wheat dataset combining the information from a previous study [14], were implemented in the analysis. The field data (FHB ratings) from rye and wheat could be combined because both hosts were inoculated with the same populations of *F. culmorum* in the same locations in one year (2015). Therefore, in the analysis of this Data set, we added a crop effect to the model.

We estimated variance components using the linear mixed model:

- Data set 1: Rye 2015 + 2016 across 2 locations per year
y*_ijn_* = μ + Iso*_i_* + Year*_j_* + Loc*_k_* + (Year × Loc)*_jk_* + (Year × Loc × Rep)*_jkn_* + (Iso × Year)*_ij_* + (Iso × Loc)*_ik_* + (Iso × Year × Loc)*_ijk_* + (Year × Loc × Block)*_ikm_* + e*_ijkmn_*, (1)
- Data set 2: Rye & wheat 2015 across 2 locations
  y*_ijn_* = μ + Iso*_i_* + Crop*_l_* + Loc*_k_* + (Crop × Loc)*_lk_* + (Crop × Year)*_jk_* + (Crop × Loc × Rep)*_lkn_* + (Iso × Crop)*_il_* + (Iso × Loc)*_ik_* + (Iso × Crop × Loc)*_ilk_* + (Crop × Loc × Rep × Block)*_iklm_* + e*_ilknm_*, (2)

where y*_ijn_* is the aggressiveness of the *i*th isolate in the *j*th year at the *k*th location, *m*th block and *l*th crop. Iso, Loc, Rep and e*_ilknm_* denote isolate, location, replication or their interactions and the residual error, respectively.

The variance components were estimated by applying the restricted maximum likelihood (REML) approach and their significance was verified by model comparison with likelihood ratio tests [30]. Heritability (h^2^) was estimated on an entry-mean basis as the ratio of genotypic to phenotypic variance according to Piepho and Möhring [31]. Furthermore, fixed genotypic effects were assumed to calculate the best linear unbiased estimates (BLUEs) of the genotypic values for the two Data sets (Table S1). All statistical analyses were performed with ASReml version 3.0 (VSN International Ltd., Hemel Hempstead, UK) [32].

Ten candidate genes previously found as polymorphic in our set of *F. culmorum* isolates [14] were used for this study (Table 2). For details on DNA extraction, PCR amplification, sequencing and SNP calling refer to Castiblanco et al. [14]. Finally, 97 isolates could be genotyped.

The association analysis was calculated using principal coordinate (PCo) and pairwise kinship coefficients [43] for correction of population structure. All subpopulations were grouping together in a common point cloud, only the Syrian subpopulation was partially shifted to the right [14]. A mixed linear model combining the two main principal coordinates as fixed effect and a kinship matrix for the random isolate effect was used to identify marker-trait associations in the Data sets (Table S1) [44]. The obtained *p* values were corrected for potential inflation [44]. The significance of marker–trait associations was based on a false discovery rate (FDR) and an adjusted *p* value of <0.05 as the cutoff. The proportion of genotypic variance (p_G_) explained by each SNP was derived from the sums of squares of the SNP in a linear model divided by h^2^. All calculations were done with statistical software R version 2.14.2 (The R Foundation for Statistical Computing, Vienna, Austria) [45] including packages GenABEL version 1.8 [44,46] and APE version 3.5 [47,48].

## 3. Results

FHB symptoms were successfully observed in rye after inoculation with *F. culmorum*, and large differences among the tested isolates were found as shown by the ranges (Table 3). The mean FHB rating (=aggressiveness) across the four environments (=location × year combinations) was 14.85%, varying from a minimum of 0.5% to 45%. FHB symptoms in the non-inoculated plots across the environments were not observed.

**Figure 1:**
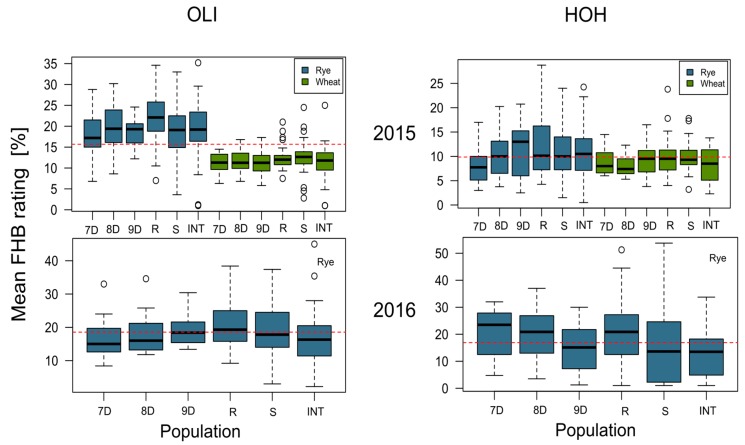
Boxplot of mean Fusarium head blight (FHB) rating (%) of five field populations (7D, Entringen, 8D, Herrenberg, 9D, Nufringen, R, Novgorod/Russia, S, Syrian transect) and the international collection (INT) of a total of 100 *Fusarium culmorum* isolates across two locations (OLI = Oberer Lindenhof, HOH = Hohenheim) in two years (2015 and 2016) and two crops (wheat, rye); the red dashed line is the grand mean across all populations, the open circles refer to outliers.

We analyzed the aggressiveness of the same 100 isolates of *F. culmorum* on wheat as a host in 2015 and on rye in 2015 and 2016 at each of two locations (Figure 1). A comparison of phenotypic data between rye and wheat is possible in 2015 where both crops were planted simultaneously in the same field and under the same experimental design. Mean FHB rating was considerably higher for rye in this year and wider ranges of aggressiveness were obtained on this crop in both locations.

**Figure 2:**
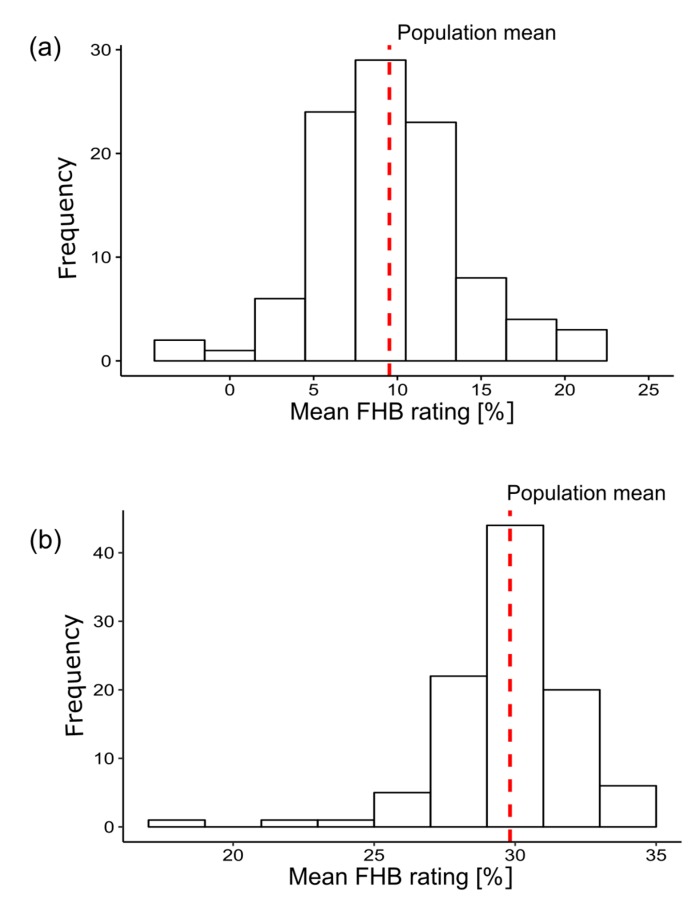
Histogram of the best linear unbiased estimators (BLUES) for mean Fusarium head blight (FHB) rating among 100 *Fusarium culmorum* isolates calculated across two years and two locations in (**a**) rye (2015 + 2016) and (**b**) wheat (2014 + 2015).

The frequency distribution of the best linear unbiased estimators (BLUES) calculated from the mean FHB rating followed a normal distribution (Figure 2) as expected for quantitative traits. The BLUES in the rye Data set ranged from −4.23% for isolate FC60 to 21.47% for isolate FC95 (Table S1). The mean across the isolates was 8.78%. In the wheat Data set the BLUES ranged from 18.92% for isolate FC60 to 34.80% for isolate S109.

**Figure 3:**
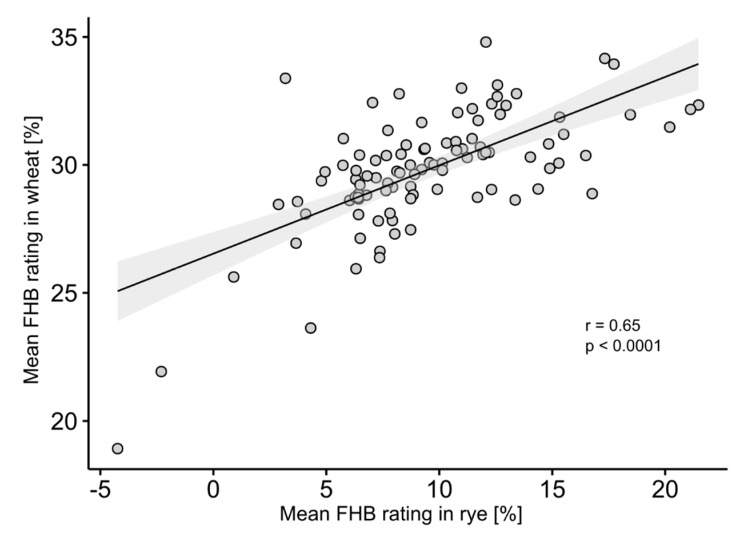
Relationship between wheat and rye calculated as BLUES with mean Fusarium head blight (FHB) rating of 100 *Fusarium culmorum* isolates across four environments; indicated is the regression line and the standard deviation (in grey); *r* = coefficient of correlation, *p* = probability of error.

The correlation of mean FHB aggressiveness on rye and wheat was significant (Figure 3).

**Figure 4:**
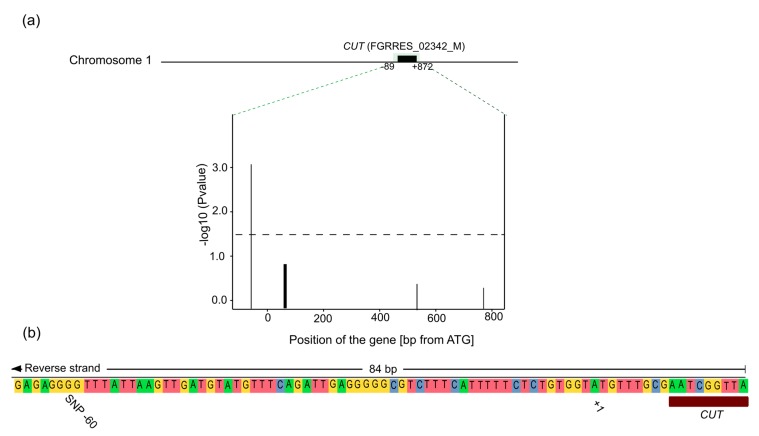
Significant association of SNPs with aggressiveness of 100 *Fusarium culmorum* isolates in the candidate gene cutinase (*CUT*). (**a**) Amplified region of the *CUT* gene on chromosome 1. The significance (−log_10_ of *p* value) of 17 SNPs was tested for this gene, each bar corresponds to one SNP. The significance by the cut-off at *p* <0.05 is shown by the dashed horizontal black line. (**b**) Location of the significantly associated SNP according to the ATG codon.

The SNP located at position −60 in the gene *CUT* (FGRRES_02342_M) was associated with field aggressiveness in both analyzed Data sets. Figure 4a shows the significance of the 17 SNP polymorphisms located in that gene, each bar represents one SNP. At position +56 to +77, 12 SNPs were closely linked resulting in a thick bar in the graph. The SNP at position −60 explained 16.05% of the proportion of the genotypic variance and was significant at *p* < 0.001.

**Figure 5:**
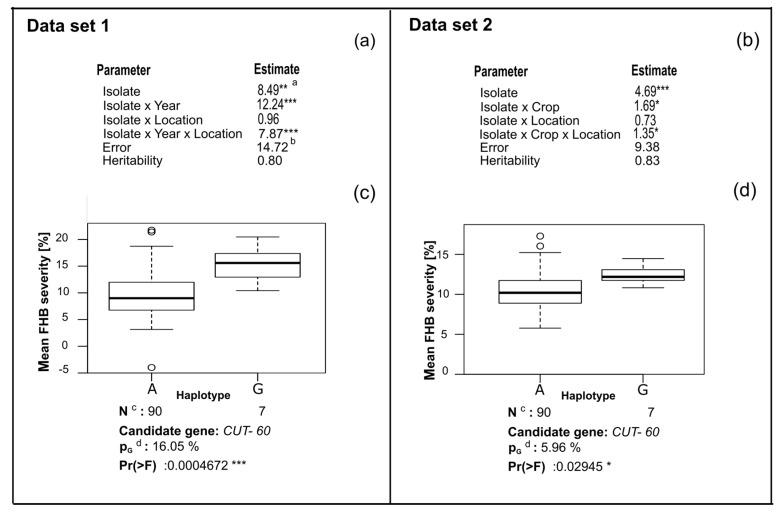
Estimates of variance components (**a**,**b**) and boxplots for mean FHB severity (%) among the two haplotypes of the associated SNP (**c**,**d**), significances and percentages of genotypic variance explained by *CUT*-*60* (p_G_) in Data set 1 (rye in 2015 + 2016) and Data set 2 (rye + wheat in 2015) after inoculation with 100 *Fusarium culmorum* isolates (Table S1). ^a^ *** Significance at *p* <0.001, ** significance at *p* <0.01, * significance at *p* <0.05; ^b^ Since heterogeneous variance for error was assumed, the reported value is the mean value of the individuals errors, ^c^ Number of isolates representing the haplotypes, ^d^ Percentage of the genotypic variance explained.

The variance components were estimated for Data set 1, which corresponds to two years and two locations in rye (Figure 5a). The isolate variance was significant (*p* < 0.01) for mean FHB aggressiveness. The isolate-by-year and the three-way interaction variances were also significant (*p* < 0.001), isolate-by-location interaction variance was not important. The entry-mean heritability for Data set 1 was 0.80.

When aggressiveness measured during 2015 on rye and wheat was combined (Data set 2, Figure 5b), there was a smaller, albeit significant, isolate variation than in Data set 1. The isolate-by-crop and the three-way interaction variances were small and significant only at *p* <0.05. The genotype-by-location interaction variance was not significant in this analysis and the entry-mean heritability was 0.83.

The two haplotypes found for the associated SNP had a significantly different aggressiveness with the isolates having the SNP with the minor allele frequency being more aggressive in both Data sets (Figure 5c,d). The percent of explained genotypic variance was considerably larger for Data set 1 than for Data set 2 (16.05% vs. 5.96%).

## 4. Discussion

Fusarium head blight is a disease with global relevance since it causes large economic losses and harmful mycotoxin contamination of the grain. In contrast with the numerous investigations on the genetics of quantitative resistance to FHB in cereals, studies on the genetic basis of aggressiveness components in *Fusarium* and other fungi are limited. Increasing our knowledge of the genetic mechanisms by which pathogens damage their hosts is of particular importance for the efficient protection of cultivated host plants and may allow us to monitor pathogenicity pathways necessary for fitness or adaptation.

Even though the importance of genome-wide association studies (GWAS) has increased in recent years, candidate gene association studies allow a direct identification of genes, which play a role in the performance of the pathogen population, even when the genome information is still scarce [49]. This methodology has helped in the detection of genes for important traits in different organisms, such as maize [50,51], rice [52], wheat [53], Arabidopsis [54], and humans [20]. Moreover, this approach was successfully used to study aggressiveness and mycotoxin production in *Fusarium* species in wheat [14,21]. In this study, candidate gene association mapping was performed for *F. culmorum* aggressiveness in rye and compared with the outcome of a previous similar study in bread wheat [14]. From an international collection, 100 *F. culmorum* isolates were used to estimate the association of ten candidate genes, previously reported to be involved in pathogenicity (Table 2) with field aggressiveness.

### 4.1. Analysis of Phenotypic Data

*F. culmorum* populations displayed a high genotypic variance of field aggressiveness within individual field populations, similar to the variance displayed by the international collection (Figure 1). This pattern has been reported in other studies with winter rye seedlings inoculated with *F. culmorum* field populations in the greenhouse [55] and with wheat adult plants inoculated with *F. graminearum* in the field [56]. This high genetic variation allows phytopathogens to adapt quickly to new conditions such as a resistant crop or changing environments [57]. The high variability of the *F. culmorum* populations increases their evolutionary potential, which is important to consider when developing successful control strategies [57,58].

In the analysis of the two datasets analyzed for FHB aggressiveness of 100 isolates, high heritabilities and significant (*p* < 0.01) isolate effects were obtained. Heritability is used in plant breeding as an indicator of the precision of the trials or a series of trials and for partitioning the total variance into the genetic and non-genetic components [31]. The mean heritability of the two datasets was 0.82, which is similar to a previous study with 42 *F. culmorum* isolates in winter rye, where the heritability value was 0.85 [59]. Significant quantitative isolate variation has previously been reported for aggressiveness studies of *F. graminearum* [26] and *F. culmorum* populations [14]. These results taken together allow the conclusion that the isolates used in this study displayed wide and consistent genetic differences in aggressiveness, which were systematically observed across a series of multi-environmental field trials. 

When only the rye data were analyzed, corresponding to the years 2015 and 2016 (Data set 1), all interactions with isolate and year were significantly (*p* < 0.001) different from zero (Figure 5). This result is consistent with the contrasting weather conditions during both years. In 2015, the relative humidity was lower compared with other years, the total rainfall was 20% less than in 2016 and these differences were even larger if the rainfall patterns are compared during the experimental period. Accordingly, lower means and ranges of aggressiveness of the *F. culmorum* isolates under study were observed for both crops in 2015 (Figure 1). In contrast, 2016 was particularly favorable to fungal infection and disease development. In quantitative pathosystems, significant interactions with the environment are commonly reported [60,61,62]. The fact that the isolate-by-year interaction played a crucial role on the expression of field aggressiveness, but not the isolate-by-location interaction suggests that trials with different years must be used in order to get reliable results when testing for pathogen aggressiveness.

Previous studies have addressed whether an isolate-by-host genotype interaction exists by using a few pathogen isolates on different host genotypes of one particular crop. Some of those studies have reported very low or lack of isolate-by-host interaction [63,64] and therefore no race specificity [65] in *F. culmorum* and *F. graminearum*. In contrast, other researchers have detected a significant interaction [66,67], but the authors argue in the discussion that the aggressiveness of isolates largely varied and the significance was rather produced by scaling effects [67]. Taking all studies together, we find contradictory and inconclusive results. The analysis of the Data set 2, which involved the comparison of the aggressiveness for the *F. culmorum* population in rye and wheat, revealed only a small, although significant (*p* < 0.05), isolate-by-crop interaction. Accordingly, the correlation between the aggressiveness of isolates for wheat and rye was significant (*r* = 0.65, *p* < 0.0001) i.e., the isolates ranked similarly on both crops (Figure 3). Despite the horizontal nature of Fusarium resistance, the significance in isolate-by-crop interaction should be examined in more detail in future because it might reflect changes in the dynamics of pathogen evolution in different cereal crops. Whether those changes are a hint for the beginning of a pathogenic specialization process as a product of the selection pressure imposed by agricultural ecosystems should be properly analyzed [68].

### 4.2. Candidate Gene Association Mapping

The sequence of the *F. culmorum* genome is still under development. Currently, two groups are working on it. Firstly, there is a fragmented assembly of an Australian strain CS7071 isolated from wheat crown rot (unpublished, Genebank accession CBMH010000000). The second group recently presented a draft assembly for a British strain (UK99) from an infected wheat ear [69]. For the present study, the annotated *F. graminearum* genome sequence and the high homology between these two *Fusarium* species were exploited [70].

The SNP-60 in the *CUT* gene displayed significant association to FHB aggressiveness and was still significant after correction for population structure with a kinship matrix coupled by a principal coordinate analysis (PCoA). Using Data set 1, which involves the aggressiveness measured on rye alone, the SNP *CUT-60* explained 16.05% of the genotypic variance with a *p*-value of 0.001. In Data set 2 which analyzed data from wheat and rye, the genotypic variance explained by the SNP was 5.96% only with *p* <0.01. Clearly, rye alone had a larger effect on this SNP than rye and wheat together.

Usually, susceptible plant genotypes allow the expression of larger aggressiveness differences when exposed to different pathogen isolates. In this study, the variability of aggressiveness expressed by the *F. culmorum* population was larger in rye than in wheat in 2015 (Figure 1), although rye is usually less susceptible to FHB than wheat [71,72,73]. This result is attributed to the characteristics of the selected experimental rye genotype combined with favorable weather conditions in 2015. Rye used for commercial production represents mainly complex hybrid cultivars that are phenotypically heterogeneous and genetically highly heterozygous. In order to measure reliable differences of isolate aggressiveness, a genetically homogeneous plant genotype was required. For the purpose of the presented research, a rye F_1_ single cross between two inbred lines (A × B) was designed, which was genetically homogeneous and more susceptible than the commercial rye cultivars. The wheat genotype used for comparison was the moderately susceptible line cultivar “Inspiration”. Consequently, the aggressiveness variation in rye was larger than in wheat in 2015.

Among all the candidate genes tested, *CUT* was the gene having most SNPs with a minor allele frequency (MAF) >5% (Table 2). The isolates that present the less common allele of the associated SNP displayed on average higher aggressiveness values (Figure 5c,d). The allele frequencies of the associated SNP can give a hint of the type of selective forces influencing the trait. Since the SNP with a minor allele frequency of 0.07 at *CUT-60* represents an advantage for the pathogenic development of the fungus, it could be under positive selection and a recent selective sweep at this locus might explain the existence of rare alleles [74]. However, it cannot be ruled out that the significant polymorphism associated with aggressiveness could be in linkage disequilibrium (LD) with the causative SNP [75] present in the upstream region of the *CUT* gene that was not sequenced.

### 4.3. CUT Gene Is Associated with Aggressiveness in Rye

*CUT* was significantly associated with FHB aggressiveness and showed high nucleotide diversity. Comparative genomic studies have shown that genes involved in niche adaptation, such as the colonization of living plant tissue, appear to have a high diversity among isolates of the same *Fusarium* species [70]. Cutinase is an enzyme produced by several fungi and bacteria. It is a serine esterase that catalyzes the hydrolysis of cutin into fatty acid monomers. Basically, cutin and waxes are the major structural components of the plant cuticle [76], but the arrangement and composition of the cuticle varies largely among plant species, development stages and plant organs [77]. The cuticle is a shielding membrane of the aerial segments of plants such as non-woody stems, leaves and fruits, creating the first physical barrier that phytopathogens have to overcome and it is a source of nutrients for saprophytes.

In the field of host-pathogen interactions, different functions have been attributed to the cuticle: spore attachment [78] and host signaling [79]. The penetration process assisted by cutinase has been debated for many years [80]. This role of cutinase was proved in some studies [81,82] and rejected by others [83]. In *F. culmorum* and *F. graminearum*, an active route for colonization is the invasion of the cuticle and cell wall with short hyphae [84,85]. Disruption of the cuticle was detected in a cytology study performed after *F. culmorum* inoculation on wheat [84]. The direct role of cutinase in this process, however, has not been proved yet. The *F. graminearum* genome preserves diverse cutinase genes [17] and 32 up-regulated genes, predicted as plant cell-wall degrading enzymes, among them cutinases, were identified in a gene expression study of *F. graminearum* during infection on barley heads [17]. Accordingly, it was shown most recently, that a *Verticillium dahliae* extracellular cutinase (*VdCUT11*) is an important secreted enzyme affecting aggressiveness in *Nicotiana benthamiana* [86].

Based on this information and the results in this study, we hypothesize that variations in *CUT* regulation may influence the capacity of *F. culmorum* to penetrate the host in its initial biotrophic phase or may help in its saprophytic phase. Some authors suggested an essential role of the protein during saprophytic development [87].

A model was proposed in which cutin monomers that result from the action of *Fusarium* sp. cutinases stimulate host defense responses by creating a complex with plant nonspecific lipid transfer proteins (nsLTPs), and thus facilitating cutin repair [88]. It is not known whether the *CUT-60* polymorphism associated with aggressiveness produce a decrease or increase in the cutinase expression. Under this defense model of the host and an evolutionary scenario, e.g., host evasion from *Fusarium* sp., low expression of the enzyme could delay the recognition by the host defense system and thus increase aggressiveness.

The cuticle could have a role in the plant defense by influencing the deposition of inoculum in the initial stages of infection. Waxes on the plant surface can repel water and therefore prevent the formation of a water film that the pathogen needs to germinate. The role of the cuticle as a mechanical barrier is still not clear. In pathogens that enter the host plant only by direct penetration, a thick cuticle could increase resistance to infection. *F. graminearum* and *F. culmorum*, however, can enter passively by innate openings, such as stomata, or actively by direct penetration, where the plant cuticle still may play a role in resistance. Yoshida et al. [89] evaluated the relationship between FHB resistance in barley and different traits, among them wax coating. According to their results, the wax coating might have a small effect to reduce FHB infection. The authors hypothesize that this could be due to water repellency of the spike.

Interestingly, the gene *HOG1* previously reported as associated with *F. culmorum* aggressiveness in wheat as host [14] was not significantly associated with aggressiveness when rye was used as a host plant. One reason why no SNP within *HOG1* was significantly associated with aggressiveness, although there was nucleotide diversity also in rye, might be that the effect of the SNP is not stable across environments. QTL-by-environment interactions are typical for quantitative traits [90]. Some QTL vary in the magnitude of their allelic effects or they are active in certain environments but not in others. These interactions are possible mechanisms that preserve the genetic variation of quantitative traits in the population [91]. On the other hand, the association study of aggressiveness for *F. culmorum* in wheat [14], although it revealed one SNP in the *CUT* gene (*CUT* position 536 + 1) significant at *p* <0.1, did not display any significance with *CUT-60*. Given the nature of the cutinase protein, codified by the *CUT* gene of *F. culmorum*, a possible explanation for the differences could be due to differences in the cuticles of wheat and rye. Cuticles vary significantly in their architecture, for example changes in thickness according to the species and ontogeny [92]. There are differences between wheat and rye in the epicuticular wax layer: Rye ears, stems and leaves look gray and those of wheat green. The thick, gray waxy layer of rye can be easily rubbed off illustrating that it is really wax [93,94]. Therefore, it might be no surprise that the fungal cutinase has a larger impact in rye than in wheat. Accordingly, Harris et al. [95] observed in a transcriptomics study four days after infection host-specific gene expression among wheat, barley, and maize as hosts of *F. graminearum*.

### 4.4. Location of the SNP

The associated polymorphism was located −60 bp upstream from the start codon in the *CUT* gene (Figure 4b) and is caused by a change in the base pair G/A. One possible explanation for the identification of an SNP associated with aggressiveness and located in the upstream non-transcribed region of the gene could be that the SNP is in LD with the “real” polymorphism responsible for the trait variance, in closely located genes or in another region of the *CUT* promoter that was not sequenced. Another explanation might be that the polymorphism is in fact located within the promoter region, given that those regions are normally directly adjacent to the gene. Therefore, a change in this region could influence the gene transcription levels. Regulation elements of the cutinase gene promoter have been identified in the upstream non-transcribed region of the cutinase gene in *F. solani* f.sp. *pisi* [96]. According to this study, the effects are manifold: Firstly, a silencer between −287 and −249 bp from the ATG codon keeps basal gene expression low and affects the inducibility of the gene. Secondly, an antagonist of the silencer at −360 and −310 bp was detected. Thirdly, mediated basal transcription is located within first 141 base pairs of the cutinase promoter. Finally, there is a GC-rich palindrome at −171 bp, which forms the binding site of cutinase transcription factor CFT1.

## 5. Conclusions

This study demonstrates the potential of candidate gene association mapping to reveal genes that affect fitness traits for populations of plant-pathogenic fungi under field conditions. This approach is an alternative to traditional QTL studies, especially when recombinant mapping populations are not available. Natural field populations of *F. culmorum* possessed a high genetic diversity in aggressiveness that enables the infection process and increases FHB damage. The identified cutinase gene should be further analyzed by gene expression studies to validate its importance in *F. culmorum* aggressiveness using different cereal hosts including rye. Whole-genome sequencing of the fungus in future will enable a verification of our association mapping study, allow detection of more genes relating to aggressiveness and improve our understanding of the genetics that contributes to this important, quantitatively inherited trait. 

## Figures and Tables

**Table 1:** Population name, number of isolates, origin, host and year of sampling of the *Fusarium culmorum* populations used for inoculation.

Name	No. of Isolates	Origin	Host	Year of Sampling
7D	10	Entringen, Germany	Winter Wheat	2008
8D	12	Herrenberg, Germany	Winter Wheat	2008
9D	11	Nufringen, Germany	Winter Wheat	2008
R	19	Novgorod, Russia	Winter Wheat	1994
S	26	Coastal mountains, Syria	Spring wheat	2007
INT	22	International	Different cereals	1952–1995

**Table 2:** Candidate genes under study and number of SNPs with minor allele frequencies >5% [14].

Rres v4.0 Annotation ^a^	Gene	No. of SNPs ^b^	Function and References
**Genes encoding transcription factors**			
FGRRES_08811	*EFTU*	1	Elongation factor 1α elicits an immune response in the host (Pathogen Associated Molecular Pattern, PAMP) and was identified as differentially secreted [33]
**Genes encoding proteins involved in signal transduction**			
FGRRES_06878	*CMK1*	1	Predicted virulence associated protein [34], probable CMK1/2 protein kinase type I [35]
FGRRES_16491	*STE11*	1	Belongs to MAPK module regulating fungal development and pathogenicity in *F. graminearum* [36]
FGRRES_08531	*Erf2*	1	Associated with aggressiveness [21]
FGRRES_09612	*HOG1*	3	Regulates hyphal growth, stress responses and plant infection in *F. graminearum* [37]
FGRRES_16251	*TRI6*	2	Global transcription regulator in *F. graminearum* associated with affected severity in *F. culmorum* [38]
**Genes encoding membrane proteins**			
FGRRES_05633	*MSB2*	3	Transmembrane sensor that regulates invasive growth and plant infection in fungi [36,39]
**Genes encoding secreted proteins**			
FGRRES_02342_M	*CUT*	17	Predicted cutinase, required to penetrate the host cuticle [33]
FGRRES_05906	*FGL1*	4	Secreted fungal effector lipase [40,41]
FGRRES_00838	*HSP70*	1	Involved in heat-shock response and found to be secreted differentially under pathogenicity conditions in *F. graminearum* [33]

^a^ The given ID (FGSG) is the entry number of the Rres v4.0 annotation *F. graminearum* genome database [42]; ^b^ SNPs detected among the 100 isolates of *F. culmorum* analyzed in this study.

**Table 3:** Means and isolate ranges of mean Fusarium head blight (FHB) rating of rye after inoculation with 100 *Fusarium culmorum* isolates at two locations in two years (four environments).

Environment	Mean FHB Rating	
	Mean (%)	Isolate Range (%)
2015—Hohenheim	11.01	0.50–32.25
2015—Oberer Lindenhof	19.36	1.00–35.20
2016—Hohenheim	11.16	0.66–41.66
2016—Oberer Lindenhof	17.88	2.20–45.00
Combined	14.85	0.50–45.00

## Supplementary Materials

The following are available online at www.mdpi.com/2309-608X/4/1/14/s1. Table S1: Best linear unbiased estimates (BLUEs) for 100 *F. culmorum* isolates in rye (Data set 1) and wheat+rye (Data set 2).
