# Supplementary material for: Candidate Genes for Aggressiveness in a Natural Fusarium culmorum Population Greatly Differ between Wheat and Rye Head Blight
Source: J Fungi (Basel). 2018 Jan 16;4(1):14. doi: 10.3390/jof4010014 (PMC5872317; doi:10.3390/jof4010014)
Supplement: Supplementary file 1 [file jof-04-00014-s001.pdf]

Table S1. Best linear unbiased estimates (BLUEs) for 100 *F. culmorum* isolates in rye (Data set 1) and wheat/rye (Data set 2).

Name	Mean FHB (Data set 1)	Mean FHB (Data set 2)
7D22	6.02996208	7.026035841
7D23	4.783959899	9.32667303
7D24	9.567929844	11.40888751
7D26	4.085511852	6.272004522
7D27	6.305742364	7.792814215
7D28	7.0472762	12.02333653
7D34	8.717224372	10.7144464
7D6	11.02548582	9.541806156
7D7	9.762118551	12.11685281
7D9	13.35986824	10.24860952
8D13	7.932579076	8.196516495
8D14	7.371765296	6.246690897
8D17	7.202349919	8.622956021
8D2	11.45239205	12.67311073
8D20	10.1355119	9.96455015
8D28	6.80660376	9.273322832
8D3	14.8928292	11.8164075
8D33	6.432052481	9.928018495
8D4	8.123812245	10.88568785
8D5	15.29040787	13.46829237
8D6	8.736143272	10.59995312
8D8	11.81896909	11.93197962
9D1	7.718832191	9.132302991
9D11	6.778704587	10.24857189
9D18	8.841593314	8.821060701
9D22	10.32627843	13.15177003
9D31	6.46895175	7.176876878
9D32	12.69862297	13.27772719
9D34	12.21168898	10.79381433
9D37	9.220184249	9.408678217
9D38	7.925836708	9.336535945
9D40	9.22230271	12.29534403
9D5	12.95725778	13.35976826
FC104	11.71981413	15.55422342
FC106	7.301953639	9.763324609
FC2	15.50671146	13.69555682
FC3	12.32412826	13.16894999

Name	Mean FHB (Data set 1)	Mean FHB (Data set 2)
FC33	11.46376967	14.03612338
FC37	8.733090228	9.814494185
FC40	8.252606321	11.1535056
FC46	12.31325909	13.35240756
FC50	4.309048539	6.20885782
FC60	-4.237592853	0.094455534
FC65	3.190563794	11.26880413
FC68	6.314777244	7.933894896
FC69	8.736990951	9.455522949
FC7	3.659595197	7.673468829
FC70	7.817520279	8.732535454
FC72	6.318132125	10.81876022
FC74	6.500164436	7.191543381
FC75	9.913760069	10.07955108
FC89	7.357148394	7.333544172
FC90	9.325131167	13.42238563
FC95	21.47313526	13.61512624
FC98	7.659554608	10.27887188
R1011	20.19923061	12.62275988
R111	9.374168944	11.95334925
R1111	5.733770882	9.902388597
R1211	7.181354638	10.53442347
R1311	11.93657778	12.57485893
R1411	12.57678783	15.65902347
R1511	4.945504711	6.928369845
R1611	8.309681833	8.601720821
R1811	14.03178312	12.16658991
R1911	16.48452245	11.78492468
R2011	12.05810596	11.25816568
R2111	10.13972981	11.25429398
R2211	6.414982181	8.339285341
R2311	15.3294195	14.9005141
R311	8.03183091	8.424443199
R411	18.45707113	16.47092361
R711	10.73321286	12.95647064
R811	17.73151451	13.97961324
R911	13.41871457	13.05674637
S021	17.32632893	13.9958185
S022	12.56616577	13.94740566
S023	8.908226892	11.15610424

Name	Mean FHB (Data set 1)	Mean FHB (Data set 2)
S043	21.12043297	13.8439849
S045	16.77046243	11.3643689
S060	2.883787399	8.50984134
S109	12.06492759	17.7207364
S129	-2.304532717	2.380355492
S220	7.725976677	10.37252396
S222	14.84108378	12.65112739
S229	5.754289743	11.49430783
S256	6.280923616	8.848624254
S259	6.489782502	9.939180571
S264	14.37590642	9.816297313
S265	7.63808642	9.689855439
S267	3.723573885	10.11695873
S274	6.426130163	11.54901996
S275	10.98428653	11.33545261
S276	10.81476726	11.35209654
S280	0.901540817	5.893721665
S283	11.68779352	12.11102087
S289	10.76439234	11.56144517
S290	11.23775162	11.7568284
S293	8.537010603	12.69128669
S296	6.4077697	10.22009255
S299	8.225054891	10.6302285
